# Experimental evidence for chemical mate guarding in a moth

**DOI:** 10.1038/srep38567

**Published:** 2016-12-09

**Authors:** Seyed Ali Hosseini, Michiel van Wijk, Gao Ke, Seyed Hossein Goldansaz, Coby Schal, Astrid T. Groot

**Affiliations:** 1 University of Amsterdam, Institute for Biodiversity and Ecosystem Dynamics, Amsterdam, the Netherlands; 2 North Carolina State University, Department of Entomology and Plant Pathology, Raleigh NC, USA; 3 University of Tehran, Department of Plant Protection, College of Agriculture and Natural Resources, Karaj, Iran; 4 Max Planck Institute for Chemical Ecology, Department of Entomology, Jena, Germany

## Abstract

In polyandrous species, males seek to maximize their reproductive output by monopolizing their mate. Often the male transfers substances to the female that suppress her sexual receptivity or antagonize the behavior of competing males; both are usually transferred in seminal fluids and represent forms of chemical mate guarding. In moths, more long-range female sex pheromones have been identified than in any other animal group, and males often display with close-range sex pheromones, yet odor-based post-copulatory mate guarding has not been described in moths so far. We tested the hypothesis that the male sex pheromone in the noctuid moth *Heliothis virescens* perfumes the female and functions as an anti-aphrodisiac. Indeed, virgin females perfumed with male pheromone extract, or with its main component, mated significantly less than control virgin females, and this effect persisted for two successive nights. This chemical mate guarding strategy was disadvantageous for *H. virescens* females, because the reproductive output of twice-mated females was significantly higher than that of once-mated females. Since the female and male sex pheromones are biosynthetically related in this and other moth species, chemical mate guarding may also impose selection pressure on the long-range female sex pheromone channel and consequently affect the evolution of sexual communication.

In many animal species, females become unreceptive after mating. This sexual refractiveness may be generated by the females themselves, e.g. through reduced emission of aphrodisiacs so that her attractiveness is reduced^1^,^2^, or through anti-aphrodisiacs that are transferred from males to females during copulation. Within the framework of sexual selection, anti-aphrodisiacs can be receptivity-inhibiting “matedness factors” that are transferred by the male in the seminal fluid and affect the female’s physiology, e.g. refs 3, 4, 5, 6, or they can be odor-based and thus perceived through the chemosensory organs of nearby males^4^.

Odor-based anti-aphrodisiacs that are deposited on the abdomen of the female have been found in both vertebrates and invertebrates^7^,^8^,^9^. Interestingly, in night-active moths where female-produced sex pheromones have been identified for >1600 species (see Pherobase.com), odor-based anti-aphrodisiacs have not been described to date. Males of some species produce a close-range sex pheromone that is emitted from their so-called hairpencils, i.e. long hairs surrounding their aedeagus^10^, whose function is still poorly understood. A function in antagonizing approaching competing males during courtship has been suggested, as for instance the hairpencils of the noctuid moth *Heliothis virescens* contain 16-carbon acetate esters, which are known repel competing males^11^,^12^.

The benefits to the signaling mated male are obvious, as prolonged non-receptivity of the female reduces sperm competition and increases the number of offspring sired by him. Receivers of this signal can be the mated female as well as competing males. The benefit for the mated female has generally been assumed to be reduced male harassment, as females are often presumed to not gain reproductive benefit from multiple matings^13^,^14^. However, the generality of this assumption is questionable, as females may mate multiply not only to increase the genetic variation of their offspring, but also to increase the females’ reproductive output^15^,^16^. Additionally, in moths, male harassment is unlikely to be significant, because females attract males only when they actively emit a long-range sex pheromone during “calling”, the active extrusion of the sex pheromone gland^17^. Hence, odor-based anti-aphrodisiacs may not be of any discernible benefits to female moths, and may even come at a cost when females do gain reproductive benefit from multiple matings.

Receivers can also be competing males, for whom the anti-aphrodisiac pheromone may be informative to distinguish virgin from mated females. This would be advantageous for receiving males if females become unreceptive after mating, so that males don’t waste energy courting unreceptive females, and/or if the chance of fertilizing a large proportion of eggs is significantly higher in virgin than in mated females. For example, in the moth *H. virescens,* females oviposit ~ half their eggs after the first mating and fewer eggs in subsequent nights^18^.

In this study, we test the hypothesis that the male sex pheromone in *H. virescens* is deposited onto the female and acts as a persistent odor-based anti-aphrodisiac. This perfuming can be regarded as post-copulatory chemical mate guarding, because the anti-aphrodisiac hairpencil compounds maximize the male’s paternity but reduce the female’s overall fecundity.

## Results and Discussion

### Males, but not females, choose virgin partners

We first determined whether males and females prefer to mate with virgin partners, and found that virgin *H. virescens* females mated equally frequently with virgin and mated males (15 virgin vs 15 mated males). In contrast, virgin males mated significantly more often with virgin than with mated females (52 virgin vs 20 mated females, χ^2^ = 10.554, d.f. = 1, *P* = 0.001). It is important to note that both virgin and previously mated females exhibited calling behavior, indicating sexual receptivity. These results show that females do not prefer to mate with virgin or mated males, but males prefer to mate with virgin females.

### Females are perfumed by males

**Figure 1:**
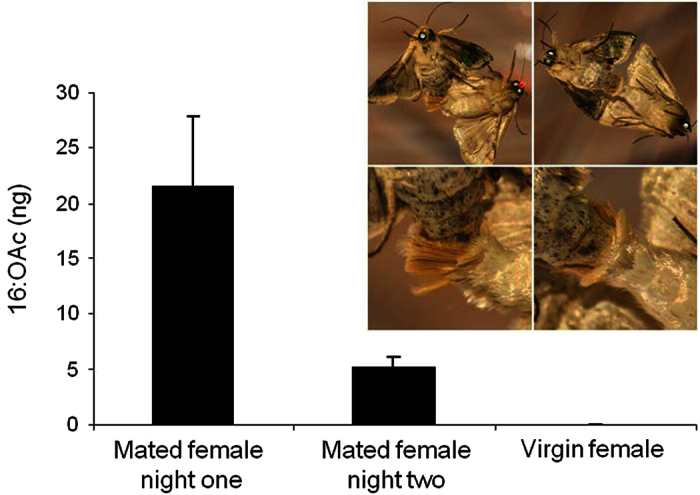
Average amount of 16:OAc (±SEM) extracted from one female thorax and abdomen. Females were extracted immediately after mating (night one, n = 13), 24–25 h after mating (night two, n = 15), or as virgins (n = 18). Insert pictures: During mating the male (on the right) envelops the terminal end of the female’s abdomen with his hairpencils.

Females extracted immediately after mating (night one), or in the second night after mating (night two), contained appreciable amounts of the major male hairpencil compound (16:OAc), whereas virgin females did not (Fig. 1). Thus, during the 2–3 h copulation, the male “perfumes” the pheromone onto the female’s abdomen by embracing the terminal segments of the female’s abdomen with his hairpencils (see photos in Fig. 1).

### Males choose unperfumed females

**Figure 2:**
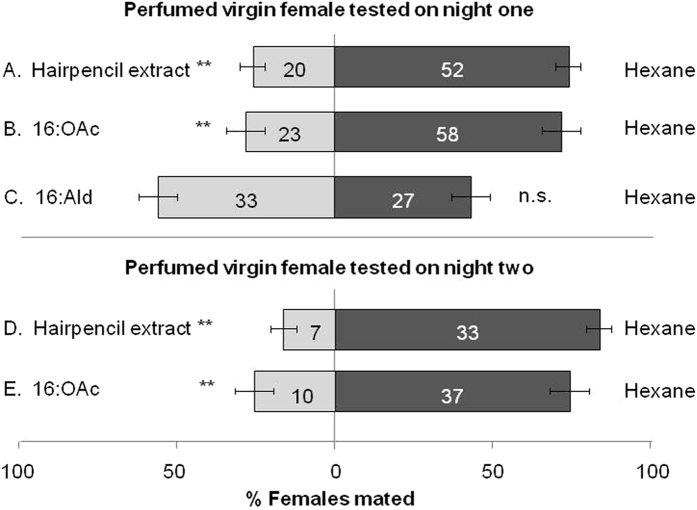
Male mate choice (±SEM) for virgin females perfumed with male hairpencil extract (**A** and **D**), the main male pheromone compound (16:OAc, (**B** and **E**)), or a compound found in the female pheromone gland (16:Ald, (**C**)). Numbers in bars are number of matings. Since we did not find differences between control females and females treated with 16:Ald, we did not test these females the second night. **P ≤ 0.0001, ns: not significant.

**Figure 3:**
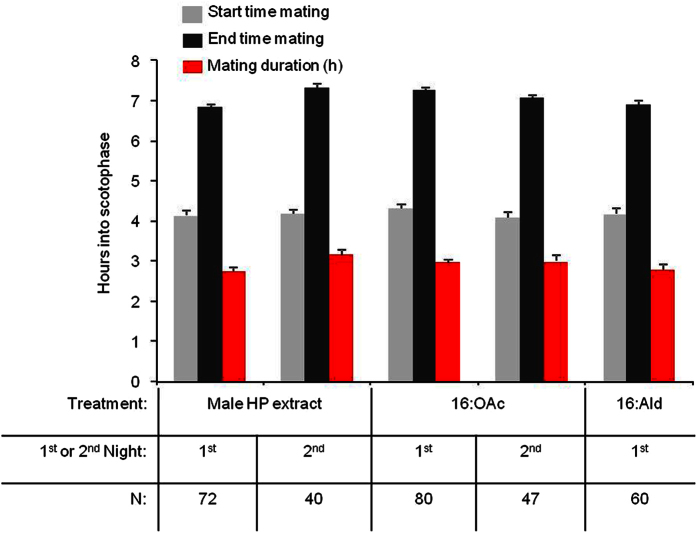
Average start and end time of all matings and the average mating duration (±SEM) of the differently treated females, showing that there were no significant differences among the groups (see text for details).

When we applied crude pheromone extract of the male hairpencils or its main pheromone component to the abdomen of virgin females, female mating chances significantly reduced (Fig. 2). This was true both for females that were tested directly after perfuming (night one) (hairpencil extract χ^2^ = 14.732, d.f. = 1, *P* = 0.0001; 16:OAc χ^2^ = 15.633, d.f. = 1, *P* < 0.0001), as well as for females perfumed in the previous night (tested in night two; hairpencil extract χ^2^ = 18.354, d.f. = 1, *P* < 0.0001; 16:OAc χ^2^ = 16.502, d.f. = 1, *P* < 0.0001). Virgin females perfumed with 100 ng of the non-pheromonal compound 16:Ald were mated as often as hexane-perfumed females (χ^2^ = 0.601, d.f. = 1, *P* = 0.4382) (Fig. 2). The start time and duration of mating did not differ between the different groups; start time mating (d.f. = 3, 282; F = 0.36, *P* = 0.77), end time mating (d.f. = 3, 282; F = 0.74, *P* = 0.52) and mating duration (d.f. = 3, 282; F = 0.25, *P* = 0.85; Fig. 3). These data suggest that the antiaphrodisiac has quantitative effects, causing a decline in mate-finding by males, but does not appear to change elements of the copulation once it is initiated. Together, these results clearly show that the close-range male sex pheromone emitted from the hairpencils of the noctuid moth *Heliothis virescens* acts as a persistent odor-based anti-aphrodisiac pheromone.

### Female fecundity is negatively affected by male perfuming

Twice-mated females tended to have a higher lifetime fecundity than once-mated females (*P* = 0.052), while the mean number of eggs per day was significantly higher (*P* = 0.012). The longevity of females and the percent hatched eggs were not affected by the number of matings (Table 1). The male strategy of perfuming females with an odor-based anti-aphrodisiac thus represents a form of chemical mate guarding, similar to what has been described in other insects e.g. ref. 19 and primates^20^.

To gain insight into the evolution of this anti-aphrodisiac pheromone, its effects on the fitness of the mated male and the main receivers – competing males and the mated female – need to be determined. In *H. virescens* the male appears to invest heavily in his mate: males mate only once per night, and one mating lasts ~3 h during which he transfers a spermatophore that is ~5–10% of his body mass^21^. Perfuming the female significantly reduces the probability that she will remate (Fig. 2). Because there is no last-male sperm precedence in *H. virescens*^22^, the perfuming strategy minimizes sperm competition and protects the male’s investment. The anti-aphrodisiac pheromone may also benefit competing males, because females oviposit ~ 50% of their eggs after the first mating and significantly fewer eggs on each subsequent night^18^. The anti-aphrodisiac pheromone may guide the competing male’s decision to abandon the calling mated female, or to pursue her but adjust his investment in her^23^.

In contrast, *H. virescens* females are negatively affected by the anti-aphrodisiac marker. The anti-aphrodisiac pheromone reduced subsequent matings and lessened female fecundity, as twice-mated females oviposited more eggs than once-mated females. Females normally re-mate every night (11 out of 21 in this study) or every other night^21^,^24^. Therefore, there may be selection pressure on females to accept males with less pheromone or less saturated pheromone components to minimize the chemical mate guarding effects, as unsaturated compounds are more volatile. Recently, *Drosophila melanogaster* females were found to actively eject the male pheromone a few hours after copulation, resulting in increased attractiveness and remating^6^. Such an active process is unlikely to occur in moths, as this pheromone is deposited on the outside of abdomen and we never observed females to actively groom her abdomen after mating. Whether and how male mating success varies with the quantity or quality of pheromone he produces remains to be tested.

Interestingly, in *H. virescens* the female long-range sex pheromone and the male close-range courtship pheromone are biosynthetically related^25^, which introduces the possibility that selection forces that shape the pheromone of one sex affect the pheromone composition of the opposite sex. If females choose to mate with males with less saturated (and thus more unsaturated) pheromone compounds, to increase the volatility and thus decrease the duration of chemical mate guarding, then there would be selection for a decreased ratio of saturated-to-unsaturated compounds. Typical *H. virescens* females produce a high ratio and are attractive to males, whereas females with a lower ratio are less attractive^26^. So far, the evolution of moth sex pheromones has been regarded as determined by selection pressures acting only on the female sex pheromones (e.g. refs 27 and 28). As these are important species-recognition cues that minimize cross-species communication interference^11^,^29^,^30^, moth pheromones are generally thought to be under stabilizing selection, which makes it hard to envision how these sexual communication systems can evolve^29^,^31^,^32^. Now that we show that the close-range male sex pheromone can also serve in both intersexual and intrasexual conflict, these additional selection pressures may help explain the enormous diversity of moth pheromone blends^33^.

In conclusion, our study shows the presence of a persistent odor-based anti-aphrodisiac in a noctuid moth, which mediates male-male competition and negatively impacts female fecundity. The major male pheromone component alone is sufficient to impose substantial chemical mate guarding of the mated female. It remains to be determined whether females choose males depending on the quantity and/or quality of their close-range pheromone.

## Methods

*Heliothis virescens* was obtained from North Carolina State University laboratory colonies, and has been reared at the University of Amsterdam since 2011 in a climate chamber at 25 °C, 60% relative humidity and a light–dark cycle of 10 L: 14 D with lights off at 11:00. Larvae were reared singly in plastic cups (37 ml, Solo, Lake Forest, Illinois) on artificial pinto bean diet^34^. Emerged adults were checked daily and provided with a 10% sucrose solution. For all experiments 2–5 day old adult moths were used.

### Do males and females choose virgin partners?

We determined whether virgin *H. virescens* males and/or females choose to mate with virgin or mated partners in 30 × 30 × 30 cm mesh cages. To obtain mated individuals, one virgin male and female were paired in transparent plastic beakers (473 ml, Solo, Lake Forest, Illinois) containing a piece of cotton soaked in sugar-water. The pairs were observed every 30 min during the scotophase. After each mating pair separated, the male and female were placed in separate beakers until the next scotophase. In the choice experiments, one virgin male or female was offered a virgin and a mated mating partner. To distinguish between virgin and mated individuals, one of the two was marked randomly with a black marker.

### Are females perfumed by males?

To determine whether females are perfumed by the male pheromone during copulation, we immersed the abdomen and thorax of a mated female directly after mating for 30 min in 150 μl hexane, containing 200 ng pentadecane as internal standard. All extracts were kept at −20 °C prior to chemical analysis and analyzed individually on an Agilent 7890 Gas Chromatograph (GC), as described in ref. 35.

### Do males recognize perfumed females?

Male choice experiments were conducted with virgin females in the same cages as described above. 30–60 Minutes before the start of the scotophase, the abdomen of each virgin female was treated with 3 μl of hexane (control) or 3 μl hairpencil extract, using a 10 μl Hamilton syringe (Reno, Nevada). Females were marked by clipping the tip of either the right or left forewing. Matings were recorded every 30 min throughout the scotophase. To assess the persistence of perfuming until the second night, when females normally resume calling, virgin females were also perfumed 6–7 h into scotophase of night one (the time that matings would generally end), and placed in cages without a male. 30–60 Min before the next scotophase (night two), virgin males were added to the cages and experiments were conducted as above. Females were perfumed with a) male pheromone extract or b) male pheromone compounds, as described below.

#### Male pheromone extract

Hairpencils of 2–3-day-old virgin males were extracted from 30 males 4–5 h after the start of the scotophase by immersing them in 1 ml hexane. 15 Min after the last hairpencils had been placed in the vial, all hairpencils were removed from the extraction vial and the extract was kept at −20 °C prior to the behavioral experiments and chemical analysis. The extract was reduced to 90 μl under a gentle flow of nitrogen, so that 3 μl represented one male equivalent.

#### Male pheromone compound

As male hairpencils of *H. virescens* contain ~ 200 ng hexadecyl acetate (16:OAc) as the main component^36^,^37^, and male approach and landing was significantly affected by exposure to 100 ng 16:OAc^38^, abdomens of virgin females were perfumed with 100 ng 16:OAc. To ensure that our findings could be attributed to this male pheromone compound and not to any odor that changes the female’s odor, we also perfumed virgin females with 100 ng hexadecanal (16:Ald). This compound is present in the female sex pheromone gland^39^,^40^ and on the male’s tarsi^41^ and perceived by both males and females^42^, but it has not been shown to affect male attraction^43^.

### Is females fecundity affected by the number of matings?

To quantify fecundity effects of a second mating on female fitness, virgin females and males were paired in the same 473 ml beakers, as described above, containing a piece of cotton soaked in sugar-water. The pair was observed throughout the scotophase, and the start time and duration of copulations were scored. Some of the mated females were paired with a new virgin male the next night and confirmed to mate again with observations throughout the scotophase. For each female, the number of oviposited eggs and hatching larvae were determined daily until death.

Statistical analysis was conducted in R-studio (0.98.490). All mate choice experiments were analyzed using GLM with a binomial error distribution. The perfumed or control females were treated as binary response variables, while the dates of the experiment were fixed effects. Cages without matings were excluded from the analysis (n = 6). To determine differences in the reproductive output of females, lifetime fecundity (number of hatching larvae) and lifespan, we first assessed normality of the data, using a Shapiro-Wilk’s normality test. Since we did not find significant deviations from a normal distribution for the reproductive output (W = 0.979, *P* = 0.7699), lifetime fecundity (W = 0.9598, *P* = 0.2712) and mean number of eggs/day (W = 0.97563, *P* = 0.6663), we used a one-way ANOVA to compare these measures between once- and twice-mated females. As the fraction of hatched eggs did deviate significantly from normality, these data were analyzed with a Mann-Whitney U-test.

## Additional Information

**How to cite this article**: Hosseini, S. A. *et al*. Experimental evidence for chemical mate guarding in a moth. *Sci. Rep.* **6**, 38567; doi: 10.1038/srep38567 (2016).

**Publisher's note:** Springer Nature remains neutral with regard to jurisdictional claims in published maps and institutional affiliations.

## Figures and Tables

**Table 1:** Fecundity and longevity (±SEM) of once-mated versus twice-mated females.

	Once-mated females (n = 21)	Twice-mated females) (n = 11)	Value test statistics	*P-*value
Lifetime fecundity	440 ± 47	543 ± 63	F = 4.016	0.051
Mean #eggs/day	30 ± 2	44 ± 5	F = 7.254	0.012
% Hatched eggs*	48.7	46.2	W = 105	0.696*
Longevity	14.81 ± 1.07	12.55 ± 1.15	F = 1.768	0.194

The twice-mated females mated on consecutive nights. Fecundity was measured by counting the number of hatching larvae of each female during her life, longevity was determined by checking each female every day until death, mean #eggs/day is the total number of eggs per female oviposited divided by longevity, and % hatched eggs is the median fraction hatched eggs of all eggs that were oviposited. All females were kept in separate transparent beakers and sugar water was replaced every other day. *Not normally distributed, and thus tested with a Mann-Whitney U-test.
